# Perforation gastrique spontanée compliquant une bronchiolite aiguë chez un nourrisson

**DOI:** 10.11604/pamj.2019.32.139.18354

**Published:** 2019-03-25

**Authors:** Aymar Pierre Gildas Oko, Patricia Irène Ondima, Marlène Kibangou Lenvo, Letitia Lombet, Judicaël Kambourou, Nelly Pandzou Guembo, Mamadou Ildevert Cyriaque Ndjobo, Aurore Mbika Cardorelle

**Affiliations:** 1 Centre Hospitalier Universitaire, Brazzaville, Congo; 2 Université Marien Ngouabi, Brazzaville, Congo

**Keywords:** Perforation gastrique spontanée, bronchiolite, nourrisson, pathogénèse, Congo, Spontaneous gastric perforation, bronchiolitis, infant, pathogenesis, Congo

## Abstract

La perforation gastrique spontanée est une affection rare chez le nourrisson et les mécanismes de survenue demeurent mal compris. Nous rapportons le premier cas congolais. Il s'est agi d'un nourrisson de sexe féminin, âgée de 5 mois qui au 4^ème^ jour d'hospitalisation pour une bronchiolite aiguë sévère associée à une diarrhée compliquée de déshydratation aiguë modérée, a présenté de façon brutale une distension importante et douloureuse de l'abdomen associée à des signes de choc. La radiographie de l'abdomen sans préparation montrait un pneumopéritoine massif. La laparotomie avait permis de mettre en évidence une perforation arrondie sur la paroi postérieure de l'estomac qui a été suturée. Les suites opératoires étaient marquées par la survenue d'un choc septique et le décès du nourrisson.

## Abstract (en)

Spontaneous gastric perforation is rare in infants and the mechanisms leading to its onset are poorly understood. This study reports the first case of spontaneous gastric perforation occurred in Congo. This case report describes a 5-month years old female infant who, on the fourth day of hospitalization for severe acute bronchiolitis associated with diarrhea complicated by moderate acute dehydration, had violent onset of severe and painful abdominal distension associated with signs of shock. Abdominal x-ray without treatment showed a large volume pneumoperitoneum. Laparotomy showed round perforation on the posterior wall of the stomach which was sutured. The postoperative course was marked by the occurrence of septic shock and by infant death.

## Introduction

La perforation gastrique spontanée est une affection grave et rare chez l'enfant particulièrement au-delà de la période néonatale [1]. Les mécanismes de survenue sont encore mal compris et demeurent un sujet à controverse [1,2]. Nous rapportons ce premier cas de perforation gastrique spontanée au Congo survenue chez un nourrisson de 5 mois hospitalisé pour une bronchiolite aiguë sévère.

## Patient et observation

Francine, nourrisson de sexe féminin, âgée de 5 mois a été admise dans le Service de Soins Intensifs Pédiatriques (SIP) pour une détresse respiratoire. Elle a présenté depuis 5 jours une rhinorrhée, une toux, une diarrhée à raison de 4-6 selles liquides par jour et une fièvre avec une température oscillant entre 37,8 et 39°c. La survenue au 5^ème^ jour d'une gêne respiratoire a justifié son admission dans le service de SIP. Il n'y a pas eu de prise d'anti-inflammatoire, ni de traumatisme abdominal. Francine est sans antécédent pathologique particulier. À l'admission, elle était consciente, modérément pâle, et fébrile à 38,2°c. On notait l'existence de signes de déshydratation modérée. La fréquence respiratoire était à 67 cycles par minute, la fréquence cardiaque à 158 battements par minute, le temps de recoloration cutanée à 2 secondes, les pouls périphériques étaient bien perçus et la saturation percutanée en oxygène (SpO_2_) à 90%. Sur le plan respiratoire, on notait l'existence de signes de lutte respiratoire très marqués, de râles sous-crépitants et sibilants diffus. L'abdomen était légèrement ballonné dans son ensemble, souple et indolore Le reste de l'examen était normal. Le bilan paraclinique (radiographie thoracique de face, CRP, l'hémogramme l'ionogramme plasmatique et la créatininémie) était normal. Le diagnostic de bronchiolite aiguë sévère associée à une gastroentérite aiguë compliquée de déshydratation modérée a été retenu. Le traitement avait consisté en une oxygénothérapie avec 3L d'oxygène par lunette, une réhydratation par voie intraveineuse avec du Ringer lactate.

**Figure 1:**
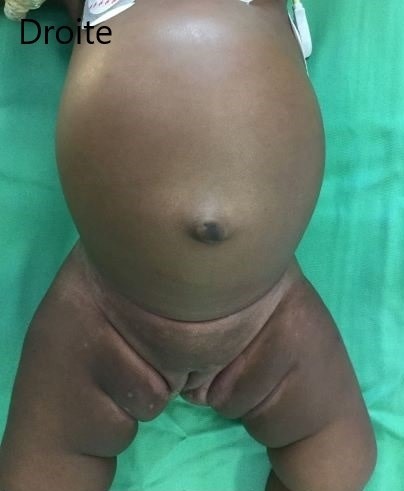
Distension abdominale importante

**Figure 2:**
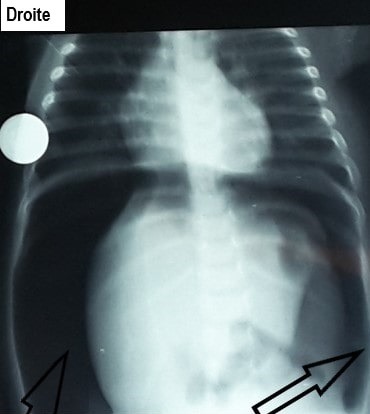
Radiographie de l’abdomen sans préparation; pneumopéritoine massif, absence de niveau hydroaérique au sein de l’estomac et une faible pneumatisation intestinale

**Figure 3:**
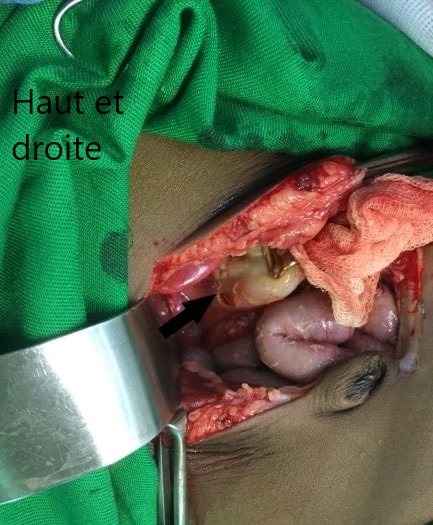
Image per opératoire de la perforation arrondie gastrique

Sous ce traitement, une amélioration progressive du tableau clinique a été observée durant les 3 premiers jours. Mais au 4ème jour, Francine était devenue léthargique, la détresse respiratoire était réapparue avec des signes de choc (en dépit de l'arrêt de la diarrhée et de la disparition des signes de déshydratation), la Spo_2_ était à 88%. Le ballonnement abdominal s'était majoré (Figure 1), l'abdomen était tendu et douloureux à la palpation et tympanique à la percussion. La radiographie de l'abdomen sans préparation (ASP) montrait un pneumopéritoine massif, une absence de la poche à air gastrique et une faible pneumatisation intestinale faisant fortement suspecter une perforation gastro-intestinale (Figure 2). Le bilan biologique montrait: une leucocytose à 14800/mm^3^c, une anémie à 8,2g/dL microcytaire, une hyperglycémie à 7,8 mmol/L, une créatininémie légèrement augmentée à 15mg/L, une légère hyperkaliémie à 4,7mmol/L, une natrémie normale à 137mmol/L. Le bilan hépatique et la mesure des gaz du sang n'ont pas été réalisés. Aussitôt une sonde nasogastrique a été mise en place et un traitement fait d'un bolus de sérum physiologique, d'une triantibiothérapie (ceftriaxone, métronidazole et gentamycine) et d'un inhibiteur de la pompe à proton a été administré. Une laparotomie a été réalisée après stabilisation de l'état hémodynamique. L'exploration a permis de trouver une perforation de forme arrondie d'environ 3,5cm x 2,5cm avec des berges propres, localisée sur la face postérieure de l'estomac (Figure 3). une suture de la perforation après ravivement des berges puis un lavage et assèchement de la cavité péritonéale ont été réalisés. Les suites opératoires ont été marquées par l'apparition d'un œdème généralisé au 2^ème^ jour, d'un choc septique au 3^ème^ jour et la survenue du décès au 4^ème^ jour.

## Discussion

La perforation gastrique spontanée est une affection rare chez l'enfant, plus observée chez le nouveau-né que chez l'enfant au-delà de la période néonatale [1]. Chez ce dernier, la perforation gastrique est souvent secondaire et peut faire suite à un traumatisme, la prise d'un médicament gastrotoxique, une gastrite ou une ulcération gastrique [2]. La perforation gastrique spontanée est une entité propre [3], seule une vingtaine de cas a été rapportée chez l'enfant après la période néonatale, il s'agissait pour l'essentiel des enfants japonais et chinois et quelques enfants européens [1]. Notre cas est le premier décrit au Congo et nous n'avons pas trouvé de cas décrit en Afrique, ce qui témoigne de l'extrême rareté de la pathologie dans cette région, mais peut être aussi de sa méconnaissance. La perforation gastrique spontanée de l'enfant au-delà de la période néonatale se distingue à plusieurs égards de celle du nouveau-né; chez le nouveau-né, plusieurs facteurs de risque ont été identifiés, parmi lesquels: la prématurité, le faible poids de naissance, l'asphyxie périnatale, l'exsanguino-transfusion, la toxémie gravidique, le diabète maternel, le placenta prævia, la chorioamniotite ou encore la rupture prématurée des membranes, il existe une prédilection pour la race noire et le sexe masculin, la perforation est souvent une déchirure linéaire localisée sur la grande courbure [4]. Tandis que chez l'enfant au-delà de la période néonatale, les facteurs de risque sont rarement présents, la pathologie prédomine chez les enfants de sexe féminin et d'origine asiatique, la perforation est souvent de forme arrondie et localisée sur la paroi postérieure ou la grande courbure de l'estomac [1]. Dans le cas présent, les caractéristiques cliniques et radiologiques, ainsi que la localisation de la perforation au niveau de l'estomac étaient globalement identiques à celles souvent décrites dans la littérature [1].

En revanche, la particularité tient au contexte de survenue, c'est-à-dire celui d'une bronchiolite aiguë sévère associée à une gastroentérite aiguë compliquée de déshydratation modérée. À notre connaissance, il s'agit d'un premier cas rapporté d'une telle association. Le mécanisme de survenue de la perforation spontanée demeure encore mal connu, plusieurs hypothèses sont évoquées: Herbut en 1943 avait émis l'hypothèse d'une agénésie du tissu musculaire gastrique, de sorte que la muqueuse est recouverte de séreuse sans muscle lisse, ainsi les ulcérations et perforations surviendraient assez facilement [5]. Mais cette théorie a été rejetée d'abord par Shaw *et al*. en 1965 puis Holgerson en 1981 qui avaient démontré grâce aux expériences sur l'estomac des chiens pour le premier et de nouveau-nés décédés pour le deuxième que l'absence du tissu musculaire à proximité de la perforation était liée à sa rétraction après perforation et non à une agénésie musculaire, et la perforation gastrique était plutôt secondaire à une surdistension gastrique [3, 4]. Ainsi la surdistension gastrique, entrainerait un étirement puis une déchirure de la paroi [3, 6]. La surdistension et l'étirement de la paroi gastrique serait aussi à l'origine de l'altération de la circulation sanguine au niveau pariétal et d'une ischémie qui favoriserait également la survenue de la perforation. L'ischémie pariétale expliquerait aussi la survenue de la perforation gastrique en situation d'asphyxie périnatale, de choc [7]. L'augmentation de la pression intraluminale gastrique lors des vomissements, toux, crises convulsives a été également citée comme cause de perforation gastrique [6, 8]. Récemment, l'équipe de Yamataka au Japon a évoqué une nouvelle hypothèse, celle d'un déficit en cellules de Cajal, cellules qui expriment un récepteur tyrosine-kinase (C-kit) et qui agissent comme un pacemaker gastro-intestinal; en effet ces cellules de Cajal sont absentes dans tous les fragments gastriques prélevés chez 3 des 7 cas de perforation gastrique et en nombre restreint dans les 4 autres cas [9].

Dans notre cas, la conjonction de plusieurs facteurs pourrait expliquer la survenue de la perforation gastrique: la détresse respiratoire peut par aérophagie occasionner une surdistension et une ischémie de la paroi gastrique, par ailleurs, l'hypoxémie observée chez le nourrisson pouvait accentuer l'ischémie tissulaire gastrique. Les quintes de toux de la bronchiolite aiguë pouvaient augmenter la pression intraluminale gastrique et contribuer à la perforation d'une paroi déjà fragilisée. Mais la perforation gastrique n'est pas une complication habituellement observée au cours des bronchiolites aiguës, un autre facteur a du contribuer à la survenue de la perforation gastrique, il pourrait s'agir de l'inflammation de la muqueuse gastrique liée à la gastroentérite aiguë ou du déficit en cellules de Cajal, malheureusement l'étude histochimique des prélèvements n'a pas été réalisée. Le tableau clinique de la perforation gastrique est assez caractéristique, Millar *et al.* ont décrit chez l'adulte 4 signes cliniques devront faire suspecter une perforation gastrique: un ballonnement abdominal important avec tympanisme, une sensibilité et tension de la paroi abdominale, un emphysème sous cutané et les signes de choc [10]. Chez notre patient, sur les 4 signes décrits par Millar, seul l'emphysème sous cutané était absent; selon Adachi *et al.* ce signe est extrêmement rare chez l'enfant [7]. A la radiographie de l'abdomen sans préparation, certains signes doivent également faire suspecter une perforation gastrique, il s'agit d'un pneumopéritoine massif, l'absence du niveau hydroaérique au sein de l'estomac et une faible pneumatisation intestinale [11]. La perforation gastrique est une affection grave, dont le pronostic a été amélioré chez l'enfant grâce aux progrès en matière de réanimation néonatale et pédiatrique [1, 6, 12]. Le pronostic dépend de la précocité et la rapidité de la mise en route d'une prise en charge appropriée, l'existence d'une acidose métabolique est un élément de mauvais pronostic [5]. Dans notre cas, la sévérité du tableau clinique et l'insuffisance de moyens thérapeutiques notamment de réanimation ont contribué au décès.

## Conclusion

La perforation gastrique spontanée est une pathologie extrêmement rare chez l'enfant en dehors de la période néonatale et potentiellement mortelle. Ce premier cas rapporté au Congo et certainement en Afrique, illustre non seulement de sa rareté mais également de sa gravité et de l'importance d'une prise en charge précoce, rapide et appropriée.

## Conflits d’intérêts

Les auteurs ne déclarent aucun conflit d'intérêts.
